# Novice Reviewers Retain High Sensitivity and Specificity of Posterior Segment Disease Identification with iWellnessExam™

**DOI:** 10.1155/2016/1964254

**Published:** 2016-01-06

**Authors:** Samantha Slotnick, Catherine Awad, Sanjeev Nath, Jerome Sherman

**Affiliations:** ^1^Private Practice, Scarsdale, NY 10583, USA; ^2^SUNY State College of Optometry, New York, NY 10036, USA; ^3^SUNY Eye Institute, Syracuse, NY 13202, USA; ^4^Nova Southeastern University College of Optometry, Fort Lauderdale, FL 33314, USA; ^5^University of Incarnate Word Rosenberg School of Optometry, San Antonio, TX 78229, USA; ^6^Eye Institute & Laser Center, New York, NY 10065, USA

## Abstract

*Introduction*. Four novices to Spectral Domain Optical Coherence Tomography (SD-OCT) image review were provided a brief lecture on the interpretation of iVue iWellnessExam™ findings (available on iVue*®* SD-OCT, Optovue, Inc., Fremont, CA). For a cohort of 126 (Confirmed) Normal, 101 (Confirmed) Disease subjects, iWellnessExam™ OD, OS, and OU reports were provided. Each novice independently reviewed and sorted the subjects into one of four categories: normal, retinal disease, optic nerve (ON) disease, and retinal + ON disease. Their accuracy is compared between the novices and with an expert reviewer.* Results*. Posterior segment disease was properly detected by novices with sensitivities of 90.6%, any disease; 84.3%, retinal disease; 88.0%, ON disease; expert sensitivity: 96.0%, 95.5%, and 90.0%, respectively; specificity: 84.3%, novices; 99.2%, expert. Novice accuracy correlates best with clinical exposure and amount of time spent reviewing each image set. The novices' negative predictive value was 92.0% (i.e., very few false negatives).* Conclusions*. Novices can be trained to screen for posterior segment disease efficiently and effectively using iWellnessExam™ data, with high sensitivity, while maintaining high specificity. Novice reviewer accuracy covaries with both clinical exposure and time spent per image set. These findings support exploration of training nonophthalmic technicians in a primary medical care setting.

## 1. Introduction

A recently published article [1] on the specificity and sensitivity of disease identification utilizing the iVue iWellnessExam™ test revealed that the data provided were sufficient for a well-trained eye clinician to review and accurately detect disease in a very high percent of subjects with either retinal and/or optic nerve (ON) disease and to accurately confirm health in an extremely high percent of healthy controls. This SD-OCT scan obtains a substantial amount of data for the assessment of both central retina and optic nerve integrity simultaneously [2–7]. (review previous study for details) [1]. A follow-up pilot study was undertaken with the same set of data to determine whether novice review of the same SD-OCT data is an effective way to identify retinal and/or optic nerve disease and to confirm health in normal subjects.

The previous study was designed to measure the specificity and sensitivity of a well-trained optometric clinician, utilizing only data obtained on the iWellnessExam™ test, in the identification of retinal and optic nerve disease in a cohort of Confirmed Normal (CN) and Confirmed Disease (CD) subjects. Specificity data were obtained by evaluating patients within the Primary Care clinic at the University Eye Center (UEC) at SUNY State College of Optometry who were determined to be both without retinal and without ON disorder (CN subjects). Sensitivity data were obtained by evaluating patients within the Ocular Disease and Special Testing Service at the UEC with known central retinal and/or optic nerve disorders (CD subjects). All glaucoma suspects were excluded from evaluation. SUNY IRB approval was obtained prior to the initiation of the study, and all subjects signed a SUNY IRB approved informed consent document.

## 2. Materials and Methods

Two groups of patients were examined: a “Confirmed Normal” (CN) cohort for the specificity aspect of the study (126 subjects) and a “Confirmed Disease” (CD) cohort for the sensitivity aspect of the study (101 subjects). Of the CD patients, 67 had retinal pathology; 50 had ON pathology. (Sixteen (16) fell into both categories, with both retinal and ON pathology.) No “glaucoma suspects” were included for evaluation, as their status as a normal or as an ON pathology subject could not be clearly established.

Data were obtained in the previous study, utilizing the iVue SD-OCT. It scans at 26,000 A-scans/second, with an axial resolution of 5 microns [8]. All analyses were made utilizing the iWellnessExam™, a one-step SD-OCT scan, which images a 7 mm × 7 mm area of the posterior pole centered on the fovea. The iWellnessExam™ report provides eight high-resolution cross-sectional retinal images, along with its data analysis results: a full retinal thickness map, a ganglion cell complex (GCC) map, and a report on Superior/Inferior (S/I) symmetry within the eye, and symmetry between eyes. Note that these scans were obtained and reviewed before the release of the normative database for the iVue system.

Four individuals who were novices at reviewing SD-OCT images were enlisted to participate in the clinical review of this data set. The novices were each of a different level of clinical and educational experience in the ophthalmic field.

*(A) Nonoptometric Technician*. This individual has served as a technician in studies involving retinal imaging technologies. He has no interest in pursuing a career in clinical optometry or ophthalmic research.

*(B) Pre-1st-Year Student/Technician*. This individual has been accepted into the professional program at the SUNY State College of Optometry. She has had 4 years of experience working in optometric and ophthalmological practices, including 18 months in an ophthalmological practice with 6 months as a technician, operating retinal scanning devices.

*(C) 1st-Year Student.* This individual was in the middle of his first year of the professional program at the SUNY State College of Optometry. Prior to entering optometry school, he spent one full year in an internship/research program, involved with the publication of unusual cases evaluated with cutting-edge ophthalmic technology.

*(D) 3rd-Year Student*. This individual was in the middle of her third year of the professional program at the SUNY State College of Optometry. She had previous experience in detecting PIL abnormalities on SD-OCT, based upon an unrelated independent study project.

These four novices were provided with a single, 1.5-hour lecture with author JS on the nature of the data obtained on iVue iWellnessExam™, and on both numerical and pictorial data interpretation. Prior to this lecture, none of the novices had any exposure to the iVue system.

Subject data sets were given a randomized code number, which served as the only identifier for each subject. Reviewers did not have access to any supplementary patient history, demographic, or clinical data. The novice reviewers were instructed to classify each subject into one of four categories: (1) normal, (2) retinal disease, (3) ON disease, and (4) retinal + ON disease. They were also requested to record the amount of time spent in review sessions so that an estimate of the amount of time spent per image could be made.

## 3. Results

Demographics and pathologies are listed in previous article [1].

**Figure 1:**
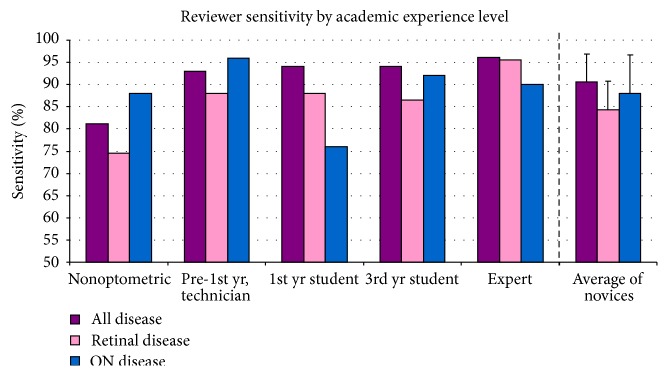
Sensitivity of reviewers for posterior segment disease identification, based on data provided with iWellnessExam™. Rightmost column set is an average of the performance of the four novices.

**Figure 2:**
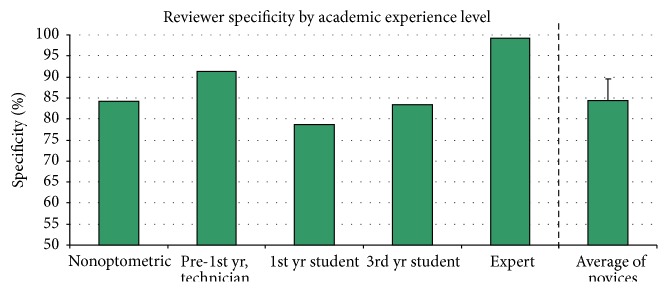
Specificity of reviewers for identification of healthy eyes among those with posterior segment disease, based on data provided with iWellnessExam™. Rightmost column is an average of the performance of the four novices.

Novice reviewers accurately identified disease (sensitivity) in 90.6 ± 6.3% of CD subjects and accurately identified health (specificity) in 84.3 ± 5.2% of CN subjects, utilizing only the iWellnessExam™ data. See Table 1 and Figures 1 and 2 for a detailed display of reviewer sensitivity and specificity data. Overall sensitivity for ocular disease improved with academic experience level.

Data were also evaluated for predictive value. These are measures of the reliability of a positive or a negative result on a test. Positive predictive value (PPV) is the percent of time that a positive test result will indicate disease. PPV is calculated as the number of true positives relative to the number of subjects who were* identified* as “positive” for the condition in question. Negative predictive value (NPV) is the percent of time that a negative test result will indicate health. NPV is calculated as the number of true negatives relative to the number of subjects who were* identified* as “negative” for any condition. (Thus, a reviewer with high sensitivity for a disease, but who tends to over-refer, will identify more subjects as positive for a test than are truly positive. This will adversely impact the PPV.)

**Figure 3:**
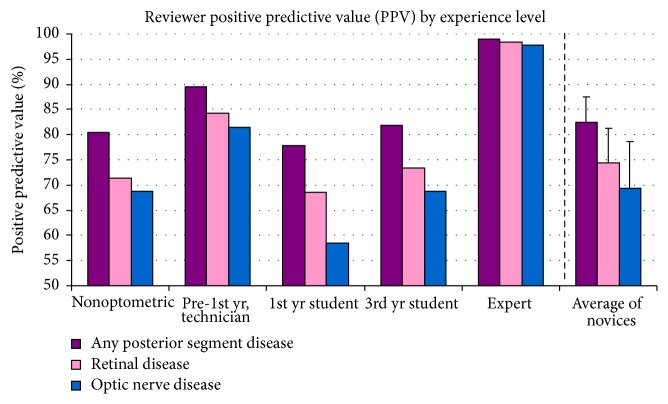
Reviewers' positive predictive value (PPV), based on data provided with iWellnessExam™. On average, novice reviewers were able to correctly predict whether a subject had disease 82 ± 5% of the time. There was a greater tendency for the novices to overrefer for optic nerve disease than for retinal disease, in all cases.

**Figure 4:**
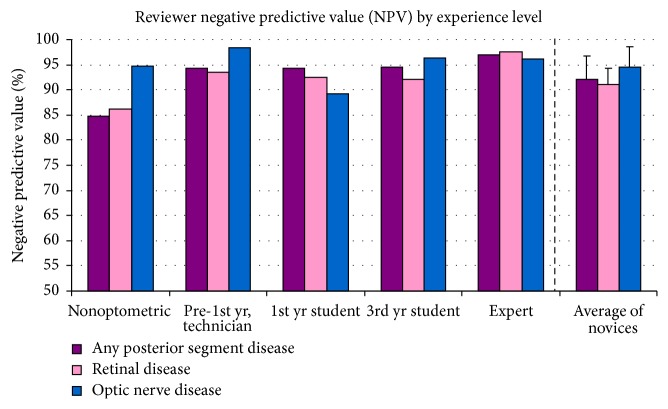
Reviewers' Negative Predictive Value (NPV), based on data provided with iWellnessExam™. On average, novice reviewers were able to correctly predict whether a subject was normal >90% of the time, with comparable performance to expert reviewer.

All novice reviewers demonstrated a greater PPV for the general category of disease than for either subcategory and a greater PPV for retinal disease than for ON disease (see Figure 3). This implies that overreferrals for disease primarily occurred in subjects who had only retinal disease (category 2) but were classified as category 4 (retinal + ON disease). The novices on the whole perform well on the most important factor: appropriate referral of patients who have any disease (82.4 ± 5.0%, with a range of 78–89%). Retinal disease overreferrals in patients with ON disease appear to abate with optometric education (3rd year more successful than 1st year at correctly identifying retinal disease). ON disease overreferral remains somewhat elevated in patients who have retinal disease. By contrast, all reviewers performed with a high NPV, ranging from 85% to 98% (see Figure 4; Table 2). If the novices identified a patient as normal, there was a 92.0 ± 4.8% chance that disease was not present.

**Figure 5:**
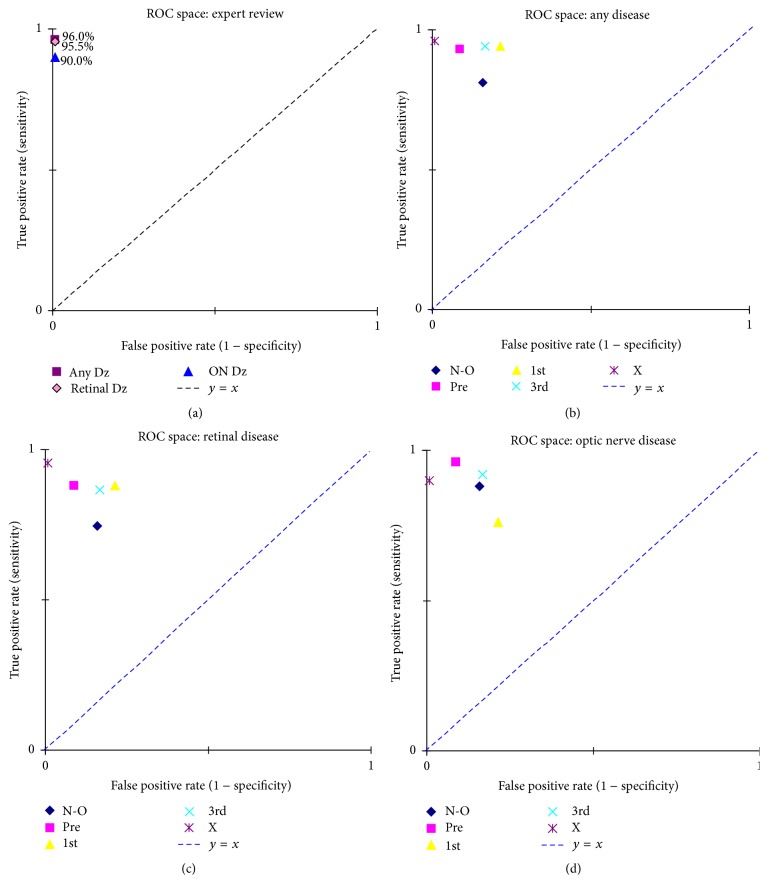
Receiver Operator Characteristics (ROC) space plots. (a) Expert performance for any disease, retinal disease, and optic nerve disease. (b–d) Comparison of all reviewers, including the expert, for (b) any disease, (c) retinal disease, and (d) ON disease. Key: N-O = nonoptometric technician; pre = pre-1st-year student/technician; 1st = 1st-year student; 3rd = 3rd-year student; X = expert. *y* = *x*: chance performance.

**Figure 6:**
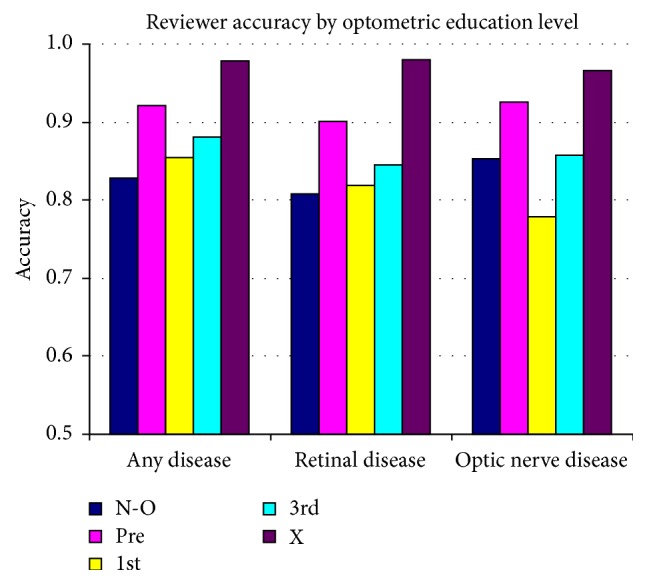
Reviewer accuracy by optometric education level.

**Figure 7:**
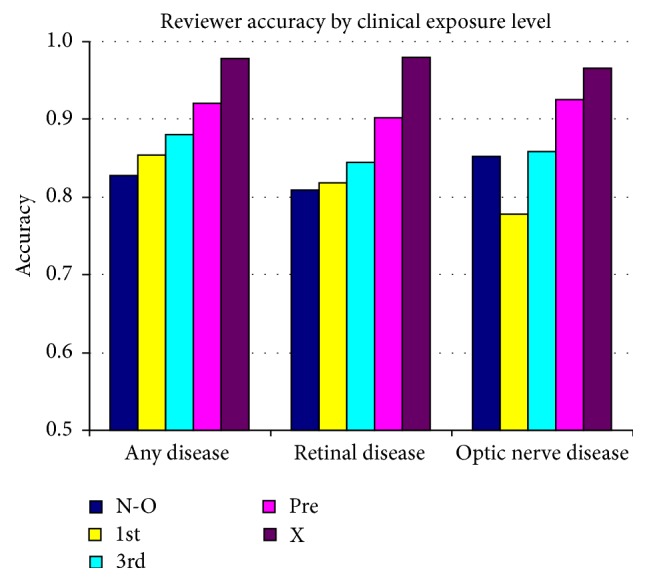
Reviewer accuracy by clinical exposure level.

With a small sample of novice reviewers, and with variations in their educational backgrounds, it is not easy to rank their relative exposures to ophthalmic conditions and expected disease identification ability. Plots of their performance were translated to Receiver Operator Characteristics (ROC) space. This evaluates each subject's false positive rate (1 − specificity) relative to their true positive rate (sensitivity). See Figure 5. Best overall performance is defined by minimizing the false positives while maximizing sensitivity, with the most desirable performance being plotted at the top left corner of the ROC space. ROC plots were used to compare (1) expert performance for overall disease and for the two subcategories of retinal and optic nerve disease (Figure 5(a)) and (2) the novices with the expert and with each other (Figure 5(b), all disease; Figure 5(c), retinal disease; Figure 5(d), optic nerve disease). For ease of comparison, the two-dimensional ROC findings are also presented as an accuracy rating. Accuracy is calculated as the sum of the true positives and true negatives divided by the sum of the total number of positives and negatives.  Figure 6 compares the novices' accuracy, arranged by relative amount of time spent in optometric education.  Figure 7 also compares their accuracy, rearranged to reflect their relative amount of clinical exposure time.

### 3.1. Time Spent per Image

**Figure 8:**
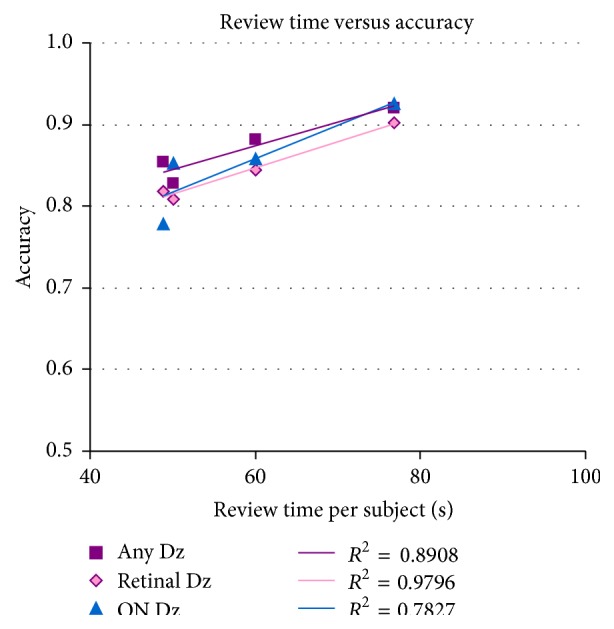
Amount of time novices spent reviewing images is compared with the accuracy of their assessments.

Novice reviewers were asked to record the time they spent performing image review. The novices conducted image review over an average of 4 sittings (ranging from 2 to 6 sittings) and spent an average of 59 ± 13 sec per image set (range 49 to 77 sec per image set). See Table 3. There does seem to be a correlation between the amount of time spent per image set and the accuracy of the subject categorization among novices (see Figure 8).

## 4. Discussion

### 4.1. Effective Screening

Above all, a screening protocol needs to be capable of disease detection. The data obtained on iWellnessExam™ may complement the clinical data obtained in the course of a routine exam [1, 9–12]. Once disease is detected or suspected, appropriate referrals can be made for follow-up testing and clinical evaluation. The results here show that individuals who are novices at reviewing SD-OCT images can be trained in a short amount of time to achieve an impressive rate of detection of the presence of posterior segment disease, while maintaining high specificity for the affirmation of health in control subjects, using only the data provided on iWellnessExam™. Another study evaluating the learning curve of a novice relative to an expert in imaging interpretation showed a similar learning effect with good accuracy when compared to the expert [13]. A study evaluating the value of problem-based learning as compared with more conventional teaching methods concludes that problem-based learning produces better educational results [14]. Thus, in a clinical environment, an ongoing feedback process between the evaluating clinician and the detecting technician will help technicians learn to interpret scans with even greater levels of accuracy.

### 4.2. Educational versus Clinical Exposure

Differences in educational versus clinical exposure are made apparent in the present pilot study. From an educational standpoint, the novices may be ranked: A < B < C < D (refer to Methods). However, from a clinical exposure standpoint, the amount of contact time with patients and with review of typical clinical data may be ranked: A < C < D < B, as the pre-1st-year technician has had* 4 years* of exposure to an ophthalmic environment and has collected clinical data from a typical cross section of the population. Assuming this technician is representative of the value of clinical learning, her performance edifies the findings of the value of problem-based learning in medical education [11, 14].

In some regards, the 1st-year student (C) may have had a relative challenge in identifying normal, as he spent a year in ophthalmic research, exposed to challenging cases of ophthalmic disease with subtle findings. This may have predisposed him to identify disease, even in subtle cases, but not to identify health (i.e., reduced specificity).

### 4.3. Interpreting Accuracy and the ROC Plots

The ROC plots enable a two-dimensional perspective on reviewer accuracy, at a glance. The Pre-1st-year optometry student who has experience as an ophthalmic technician (pink square, Figure 5) consistently outperformed the other novices. This performance supports the need for clinical exposure to general practice in order to help students contextualize their clinical observations. The 1st-year and nonoptometric reviewer have similar levels of clinical exposure. While they make different errors in reviewing the data in Figures 5(b) and 5(c) (one has more false positives with lower sensitivity; the other has fewer false positive with higher sensitivity), their performance is similar in terms of their accuracy (see Figure 7).

### 4.4. Time Invested on Image Review

The novices were asked to report on the amount of time spent reviewing the data and the number of sittings. Novices B and D took a longer amount of time reviewing each subject's data set (which consisted of 3 image files). There is an apparent correlation between the amount of time invested in image review and the accuracy of the overall categorization exercise. Interestingly, this correlation appears strongest for retinal disease (*R* ^2^ = 0.98), which requires a higher level of image scrutiny than the determination of optic nerve disease (*R* ^2^ = 0.78).

### 4.5. Challenges Predicting Optic Nerve Disease in the Presence of Retinal Disease

The reduced PPV* and* reduced sensitivity for patients with optic nerve disease, as compared to retinal disease, may be attributed to the challenges of assessing RNFL in the presence of an irregular outer retina, or even inner retinal disturbances, such as vitreoretinal adhesions. The interpretation of these challenging situations has been explored in detail, “interpreting the ganglion cell complex in the presence of retinal pathology” [1, 15].

## 5. Conclusions

The iWellnessExam™ offers the health care provider a very reliable technology for the clinical identification of eyes at risk. Novices can be trained in a short amount of time to effectively use the data from the iWellnessExam™ to screen for disease with a high rate of sensitivity, while maintaining high specificity. Accuracy of the novice reviewers covaries with both clinical exposure and time spent on image review per subject.

## Future Directions

This study shows a small sample of novice reviewers with different levels of clinical and educational exposure. It would be insightful to undertake this review with a larger sample of students at various stages of optometric education. In the interest of public health, a similar study could be undertaken with training of nonophthalmic medical technicians, to explore the potential for the identification of eye disease in patients who do not seek routine eye care but do manage their health with primary medical providers. Indeed, it is often an eye exam which results in medical referrals following the identification of retinal pathology.

## Figures and Tables

**Table 1:** Sensitivity and specificity of disease identification using only iWellnessExam™ data, by educational experience.

		Sensitivity				Specificity
		Any posterior seg. disease	Retinal disease	ON disease		Normal
Nonoptometric technician		81.2%	74.6%	88.0%		84.1%
Pre-1st yr, technician		93.1%	88.1%	96.0%		91.3%
1st yr student		94.1%	88.1%	76.0%		78.6%
3rd yr student		94.1%	86.6%	92.0%		83.3%
*Expert*		*96.0%*	*95.5%*	*90.0%*		*99.2%*

Average of novices		90.6 ± 6.3%	84.3 ± 6.5%	88.0 ± 8.6%		84.3 ± 5.2%

**Table 2:** Statistics and positive and negative predictive value (PPV and NPV).

		Any disease	Retinal disease	Optic nerve disease
PPV	Nonoptometric	80.4%	71.4%	68.8%
PPV	Pre-1st yr, technician	89.5%	84.3%	81.4%
PPV	1st yr student	77.9%	68.6%	58.5%
PPV	3rd yr student	81.9%	73.4%	68.7%

*PPV*	*Expert*	*99.0%*	*98.5%*	*97.8%*

**PPV**	**Average of novices**	**82.4 ± 5.02%**	**74.4 ± 6.86%**	**69.3 ± 9.37%**

				
NPV	Nonoptometric	84.8%	86.2%	94.6%
NPV	Pre-1st yr, technician	94.3%	93.5%	98.3%
NPV	1st yr student	94.3%	92.5%	89.2%
NPV	3rd yr student	94.6%	92.1%	96.3%

*NPV*	*Expert*	*96.9%*	*97.7%*	*96.2%*

**NPV**	**Average of novices**	**92.0 ± 4.79%**	**91.1 ± 3.32%**	**94.6 ± 3.91%**

**Table 3:** Time spent in data review, per subject (time not recorded when expert reviewed data).

	# of sittings	Time per subject (s)
Nonoptometric	6	50.1
Pre-1st yr, technician	3	76.8
1st yr student	6	48.9
3rd yr student	2	60.0

Average of novices	4.25	59.0 ± 12.9
